# Genomic Features, Comparative Genomic Analysis, and Antimicrobial Susceptibility Patterns of *Chryseobacterium arthrosphaerae* Strain ED882-96 Isolated in Taiwan

**DOI:** 10.3390/genes10040309

**Published:** 2019-04-20

**Authors:** Chih-Yu Liang, Chih-Hui Yang, Chung-Hsu Lai, Yi-Han Huang, Jiun-Nong Lin

**Affiliations:** 1 Department of Information Engineering, I-Shou University, Kaohsiung 824, Taiwan; ed101288@edah.org.tw; 2 Department of Emergency Medicine, E-Da Cancer Hospital, Kaohsiung 824, Taiwan; 3 Department of Medical Imaging and Radiological Science, College of Medicine, I-Shou University, Kaohsiung 824, Taiwan; 4 Department of Biological Science and Technology, Meiho University, Pingtung 912, Taiwan; puppylovefu@gmail.com; 5 School of Medicine, College of Medicine, I-Shou University, Kaohsiung 824, Taiwan; laich6363@yahoo.com.tw (C.-H.L.); je091410show@hotmail.com (Y.-H.H.); 6 Division of Infectious Diseases, Department of Internal Medicine, E-Da Hospital, Kaohsiung 824, Taiwan; 7 Department of Critical Care Medicine, E-Da Hospital, Kaohsiung 824, Taiwan

**Keywords:** *Chryseobacterium arthrosphaerae*, genomic features, comparative genomics, antimicrobial susceptibility, whole-genome sequence

## Abstract

Bacteria belonging to the genus *Chryseobacterium* are ubiquitously distributed in natural environments, plants, and animals. Except *C. indologenes* and *C. gleum*, other *Chryseobacterium* species rarely cause human diseases. This study reported the whole-genome features, comparative genomic analysis, and antimicrobial susceptibility patterns of *C. arthrosphaerae* ED882-96 isolated in Taiwan. Strain ED882-96 was collected from the blood of a patient who had alcoholic liver cirrhosis and was an intravenous drug abuser. This isolate was initially identified as *C. indologenes* by using matrix-assisted laser desorption ionization–time of flight mass spectrometry. The analysis of 16S ribosomal RNA gene sequence revealed that ED882-96 shared 100% sequence identity with *C. arthrosphaerae* type strain CC-VM-7^T^. The results of whole-genome sequencing of ED882-96 showed two chromosome contigs and one plasmid. The total lengths of the draft genomes of chromosome and plasmid were 4,249,864 bp and 435,667 bp, respectively. The findings of both in silico DNA–DNA hybridization and average nucleotide identity analyses clearly demonstrated that strain ED882-96 was a species of *C. arthrosphaerae*. A total of 83 potential virulence factor homologs were predicted in the whole-genome sequencing of strain ED882-96. This isolate was resistant to all tested antibiotics, including β-lactams, β-lactam/β-lactamase inhibitor combinations, aminoglycosides, fluoroquinolones, tetracycline, glycylcycline, and trimethoprim-sulfamethoxazole. Only one antibiotic resistance gene was recognized in the plasmid. By contrast, many antibiotic resistance genes were identified in the chromosome. The findings of this study suggest that strain ED882-96 is a highly virulent and multidrug-resistant pathogen. Knowledge regarding genomic characteristics and antimicrobial susceptibility patterns provides valuable insights into this uncommon species.

## 1. Introduction

The genus *Chryseobacterium*, derived from the genus *Flavobacterium*, belongs to the family *Flavobacteriaceae* [1]. The genus *Chryseobacterium* includes gram-negative, nonmotile, nonspore-forming, yellow-pigmented, oxidase-positive, catalase-positive, and rod-shaped bacteria. These microorganisms often exist in natural environments, plants, and animals, such as water, soils, rhizospheres, plants, raw milk, chicken, fish, and frogs [2,3]. Since its first introduction as a novel genus, more than 100 species have been recognized in the genus *Chryseobacterium* [4]. Among these species, *C. indologenes* and *C. gleum* are the two most common species isolated from clinical specimens [5,6]. Other species rarely cause human infections.

*C. arthrosphaerae* was initially isolated from the feces of the pill millipede *Arthrosphaera magna* Attems collected in India in 2010 [2]. Our previous study first reported four *C. arthrosphaerae* isolates obtained from patients in Taiwan [6]. We investigated these four isolates and performed the whole-genome sequencing of one *C. arthrosphaerae* strain ED882-96. In this study, we comprehensively analyze the genomic features and antimicrobial susceptibility patterns of the *C. arthrosphaerae* strain ED882-96. In addition, we compared the genomic characteristics of this strain with those of other *Chryseobacterium* species.

## 2. Materials and Methods

### 2.1. Strain ED882-96

A 39-year-old male patient presented to our hospital with chief complaints of fever and abdominal pain for two days. He was diagnosed with alcoholic liver cirrhosis and was an intravenous drug abuser. The blood culture of the patient was positive for a gram-negative bacillus. This microorganism was initially identified as *C. indologenes* by using Vitek matrix-assisted laser desorption ionization–time of flight mass spectrometry (bioMérieux, Marcy l’Etoile, France). It was named as strain ED882-96 and then stored in a glycerol stock at −80 °C.

### 2.2. Species Identification Using 16S Ribosomal RNA Gene Sequencing

After thawing from the glycerol stock, strain ED882-96 was subcultured on tryptic soy agar with 5% sheep blood (Becton Dickinson, Sparks, MD, USA). DNA was extracted using a Wizard Genomic DNA purification kit (Promega, Madison, WI, USA). Primers for the amplification and sequencing of 16S ribosomal RNA (rRNA) gene are described in Supplemental Table S1. The 16S rRNA gene sequence of strain ED882-96 was compared with sequences in GenBank of the national center for biotechnology information (NCBI) by using the basic local alignment search tool (https://blast.ncbi.nlm.nih.gov/Blast.cgi).

### 2.3. Phylogenetic Tree Based on 16S rRNA Gene Sequences

A 16S rRNA gene sequence-based phylogenetic tree was constructed using MEGA 7 [7]. The 16S rRNA gene sequences of all *Chryseobacterium* species were submitted to MEGA 7 for alignment and phylogenetic analysis based on default settings. The genetic relationships of 16S rRNA genes were inferred using the maximum likelihood method based on the JC69 model. Bootstrap analyses for 500 times were performed to provide confidence estimates for tree topologies. The phylogenetic tree generated by MEGA 7 was edited using Tree Of Life (iTOL) v4 [8]. 

### 2.4. Whole-Genome Sequencing

The genome of strain ED882-96 was sequenced using an Illumina HiSeq 4000 platform (Illumina, San Diego, CA, USA) and a PacBio RSII platform (Pacific Biosciences, Menlo Park, CA, USA). The short reads generated by the HiSeq were assembled into genome sequence using SOAPdenovo v2.04 [9]. The long reads produced by PacBio’s single molecule real-time (SMRT) detection technology were de novo assembled using the SMRT Analysis software suite 2.2 (Pacific Biosciences) [10]. The hybrid genome sequenced by the HiSeq and PacBio platforms was then assembled. The corrections of bases were performed using Pbdagcon (https://github.com/PacificBiosciences/pbdagcon), a genome analysis toolkit (GATK; https://www.broadinstitute.org/gatk/), and a short oligonucleotide analysis package (SOAP2, SOAPsnp, and SOAPindel) [11]. To trace the presence of any plasmid, filtered Illumina reads were mapped using SOAP to the bacterial plasmid database (http://www.ebi.ac.uk/genomes/plasmid.html).

### 2.5. Analysis of Genome Similarity

Whole-genome similarity was examined using average nucleotide identity (ANI) and in silico DNA–DNA hybridization (DDH). The ANI was calculated using OrthoANI [12], and an ANI value of 95% was used as the criterion for species delimitation [13]. In silico DDH was evaluated using Genome-to-Genome Distance Calculator (GGDC) (http://ggdc.dsmz.de/home.php) [14]. The recommended cutoff value for species delimitation using GGDC was 70% similarity, as examined using Formula 2 as per the recommendation of the program [13,14]. The heat maps of ANI and in silico DDH were generated using CIMminer (https://discover.nci.nih.gov/cimminer/).

### 2.6. Genome Annotation and Function Analysis of Strain ED882-96

The assembled genome was submitted to the NCBI prokaryotic genome annotation pipeline [15] and rapid annotations using subsystems technology (RAST) (http://rast.nmpdr.org/) for gene annotation and function prediction [16,17]. Orthologous genes were evaluated using clusters of orthologous genes (COGs) [18] and eggNOG [19]. Gene ontology (GO) was analyzed using InterProScan 5 [20,21]. The Kyoto encyclopedia of genes and genomes (KEGG) database was accessed to examine the high-level functions of strain ED882-96 (https://www.genome.jp/kegg/) [22]. Virulence factors were predicted using the virulence factor database (VFDB; http://www.mgc.ac.cn/VFs/) [23]. The graphical map of the circular genome was generated using CGView (http://stothard.afns.ualberta.ca/cgview_server/) [24].

### 2.7. Antimicrobial Susceptibility and Antibiotic Resistance Genes

The minimum inhibitory concentration (MIC) was examined using the broth microdilution method (Sensititre 96-well panels; Thermo Fisher Scientific/Trek Diagnostics Systems, Oakwood Village, OH, USA). Antibiotic susceptibilities were interpreted according to standards for “other non-*Enterobacteriaceae*” based on clinical and laboratory standards institute (CLSI) guidelines [25]. Because the CLSI did not include an interpretive standard for tigecycline, susceptibility to tigecycline was interpreted according to the *Enterobacteriaceae* susceptibility breakpoints of the US food and drug administration (susceptible MIC, ≤2 mg/L; intermediate MIC, 4 mg/L; and resistant MIC, ≥8 mg/L) [26]. Mutations in the quinolone resistance-determining regions (QRDRs) of DNA gyrase (GyrA and GyrB) and topoisomerase IV (ParC and ParE) were examined to determine the mechanism underlying fluoroquinolone resistance. Primers and conditions for the amplification and sequencing of QRDRs in *gyrA*, *gyrB*, *parC*, and *parE* are listed in Supplementary Table S1. QRDR sequences were aligned with those in other *Chryseobacterium* strains examined in our previous study [27]. Antibiotic resistance genes were explored using RAST [16,17], antibiotic resistance genes database (ARDB; https://ardb.cbcb.umd.edu/) [28], and comprehensive antibiotic resistance database (CARD; https://card.mcmaster.ca/) [29].

### 2.8. Type Strains of the Genus Chryseobacterium and Whole-Genome Sequences of C. arthrosphaerae

The type strains of the genus *Chryseobacterium* published in the international journal of systematic and evolutionary microbiology were obtained from the list of prokaryotic names with standing in nomenclature (http://www.bacterio.net). The 16S rRNA sequences of the type strains were explored from the GenBank of the NCBI genome sequence repository (https://www.ncbi.nlm.nih.gov/genome/). For comparison, five whole-genome sequences of *C. arthrosphaerae* available at the time of writing in the GenBank, namely ED882-96 (GenBank accession no. RYFC01000001), CC-VM-7^T^ (GenBank accession no. NZ_MAYG01000001), FDAARGOS_519 (GenBank accession no. NZ_CP033811), UBA1808 (GenBank accession no. DCFI00000000), and UBA5979 (GenBank accession no. DJBJ00000000), were downloaded for genomic analysis.

### 2.9. Comparative Analysis of COGs and KEGG

A genome-wide comparison of COGs in *C. arthrosphaerae* strains ED882-96, CC-VM-7^T^, FDAARGOS_519, UBA1808, and UBA5979 was performed using OrthoVenn (http://www.bioinfogenome.net/OrthoVenn/) [30]. Core (conserved), accessory (dispensable), and unique (strain-specific) genes of *C. arthrosphaerae* were evaluated in the COGs and KEGG by using bacterial pan genome analysis [31].

## 3. Results and Discussion

### 3.1. 16S rRNA-based Phylogenetic Relationship

**Figure 1:**
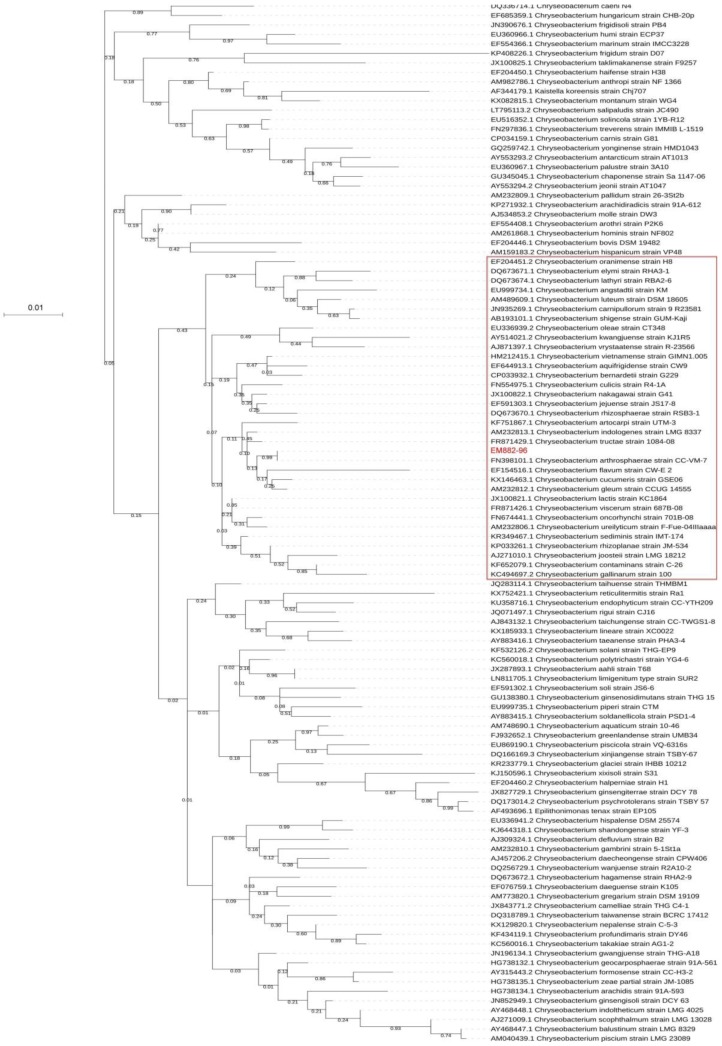
The phylogenetic analysis based on the 16S rRNA genes of strain ED882-96 and 111 type strains in the genus *Chryseobacterium*. The phylogenetic tree was constructed using the maximum likelihood method based on the JC69 model using MEGA 7. Numbers at nodes represent bootstrap values for that node based on 500 bootstrap resamplings. The phylogenetic positions of strain ED882-96 and *C. arthrosphaerae* CC-VM-7^T^ were the same. Strains in the red rectangle are those for the analyses of whole-genome similarity.

The phylogenetic tree based on the 16S rRNA of all *Chryseobacterium* species is shown in Figure 1. The 16S rRNA of strain ED882-96 was 100% identical to that of *C. arthrosphaerae* type strain CC-VM-7^T^ (GenBank accession no. NR_116977) [2]. This result suggests that ED882-96 is a strain of *C. arthrosphaerae*.

### 3.2. Basic Data of Whole-Genome Sequencing of Strain ED882-96

Two contigs and one complete plasmid sequence were assembled in the whole-genome sequencing of strain ED882-96. The statistics of assembly and annotation are shown in Table 1. The coverage for whole-genome sequencing was 309×. The assembled total lengths of the draft genomes of chromosome and plasmid were 4,249,864 bp and 435,667 bp, respectively. This whole-genome shotgun project has been deposited at DDBJ/ENA/GenBank under the accession RYFC00000000 with the version RYFC01000000.

### 3.3. Similarity of Whole Genomes

**Figure 2:**
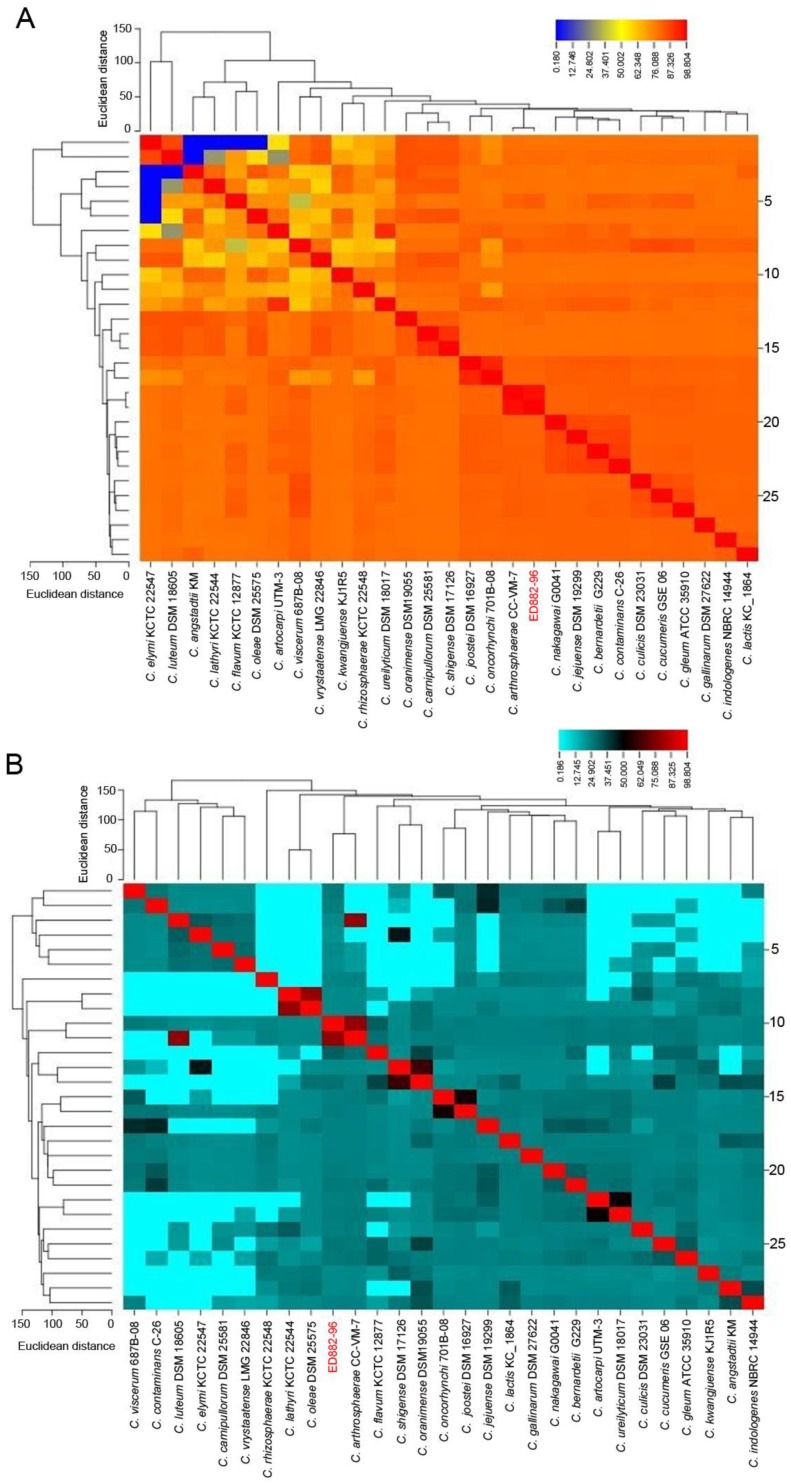
The heat maps of average nucleotide identity (ANI) and in silico DNA–DNA hybridization (DDH) between strains ED882-96 and 28 phylogenetically close *Chryseobacterium* species. (**A**) The ANI value between strain ED882-96 and *C. arthrosphaerae* CC-VM-7^T^ was 97.4%. (**B**) The in silico DDH between ED882-96 and *C. arthrosphaerae* CC-VM-7^T^ was 79.3%.

Strain ED882-96 and 28 phylogenetically close strains with available whole-genome sequences (shown in the red rectangle of Figure 1) were included for genomic analysis. The results of genomic similarity evaluated using ANI and in silico DDH are shown in Figure 2. The ANI value between ED882-96 and *C. arthrosphaerae* CC-VM-7^T^ was 97.4% (Figure 2A). The in silico DDH analysis results demonstrated that strain ED882-96 possessed 79.3% similarity with *C. arthrosphaerae* CC-VM-7^T^ (Figure 2B). The heat map of the similarity matrix clearly displayed species delineation between strain ED882-96 and other species in the genus *Chryseobacterium*. The results of ANI and in silico DDH analyses indicated that strain ED882-96 is *C. arthrosphaerae*.

### 3.4. General Genomic Annotation

**Figure 3:**
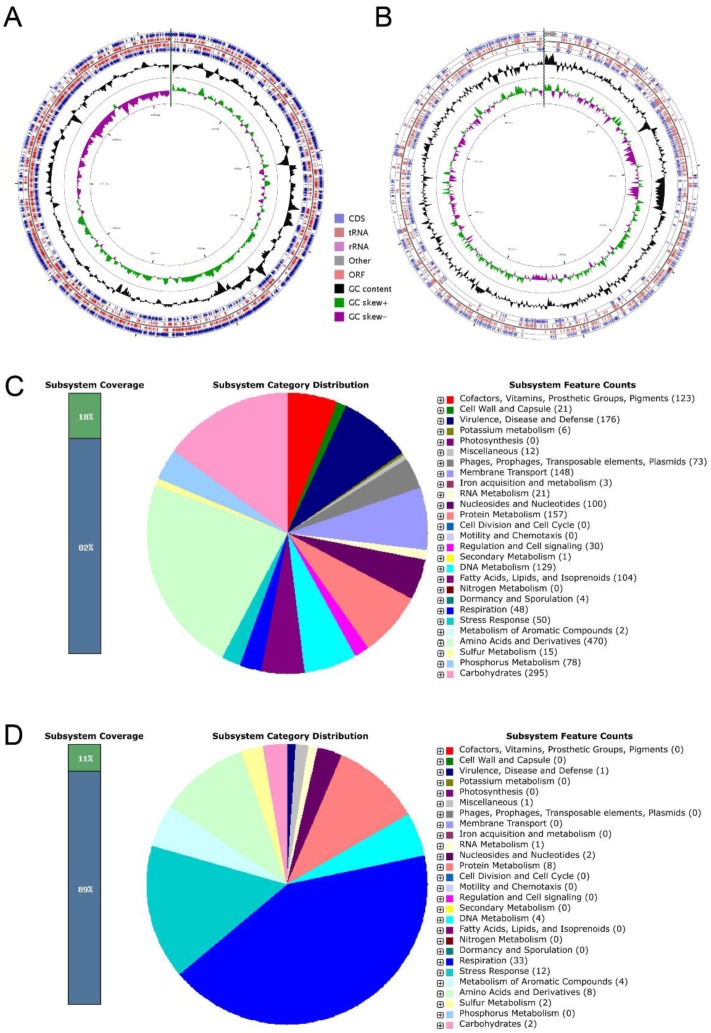
The circular representations and subsystem category distributions of the chromosome and plasmid in *C. arthrosphaerae* strain ED882-96. (**A**) The circular figure of the chromosome. (**B**) The circular figure of the plasmid. Circles are numbered from 1 (outermost circle) to 7 (innermost circle). The outer four circles show the coding sequence (CDS), transfer ribonucleic acid (tRNA), ribosomal ribonucleic acid (rRNA), and open reading frame (ORF). The fifth circle represents the GC content (black). The sixth circle demonstrates the GC skew curve (positive GC skew, green; negative GC skew, violet). The genome position scaled in kb from base 1 is shown on the inner circle. (**C**) The genome of *C. arthrosphaerae* ED882-96 annotated by rapid annotation system technology (RAST) was classified into 191 subsystems. (**D**) The plasmid in *C. arthrosphaerae* ED882-96 annotated using RAST was classified into 29 subsystems. The green part in the bar chart at the leftmost position corresponds to the percentage of proteins included. The pie chart, along with count of subsystem feature in the right panel, demonstrates the percentage distribution and category of the subsystems.

The chromosome of ED882-96 contained 5,413 coding sequences (CDSs), 6 rRNAs, and 37 transfer RNAs (tRNAs) (Figure 3A). The plasmid comprised 512 CDSs, 3 rRNAs, and 22 tRNAs (Figure 3B). The genomic features of chromosome annotated using RAST revealed that *C. arthrosphaerae* strain ED882-96 possessed 191 subsystems belonging to 27 categories (Figure 3C). Among these subsystems, “amino acids and derivatives” was the largest subsystem, followed by “carbohydrates”, “virulence, disease, and defense”, “protein metabolism”, and “membrane transport”. In the “virulence, disease, and defense” subsystem of strain ED882-96, 166 genes associated with multidrug resistance efflux pumps, multiple antibiotic resistance MAR locus, β-lactamase, streptothricin resistance, aminoglycoside adenylyltransferases, and others were recognized.

In the whole-genome analysis, “amino acid and derivatives” and “carbohydrates” were the dominant subsystems in *C. arthrosphaerae* strain ED882-96. These subsystems perform basic cellular processes and are essential to bacteria. In addition, genes that encode enzymes responsible for exopolysaccharides synthesis are characterized by soil-living organisms [32]. This finding is compatible with the fact that *Chryseobacterium* is ubiquitously distributed in soils [2,3]. Moreover, subsystems associated with “virulence, disease, and defense” were ranked third in the genome of *C. arthrosphaerae* ED882-96. This strain might be highly resistant to multiple antibiotics.

Plasmids may carry genes with different functions. In the plasmid existing in *C. arthrosphaerae* ED882-96, 29 subsystems were identified using RAST (Figure 3D). Genes associated with “respiration”, “stress response”, and “amino acids and derivatives” were the first three abundant categories among the whole subsystem of the plasmid. One “virulence, disease, and defense” subsystem that expressed β-lactamase was identified in this plasmid. These plasmid genes may play crucial roles in this strain to enhance their survival by defending against environmental factors. Further investigation of these genes is warranted to understand their accurate functions.

### 3.5. Orthologous Genes, GO, and KEGG of Strain ED882-96

**Figure 4:**
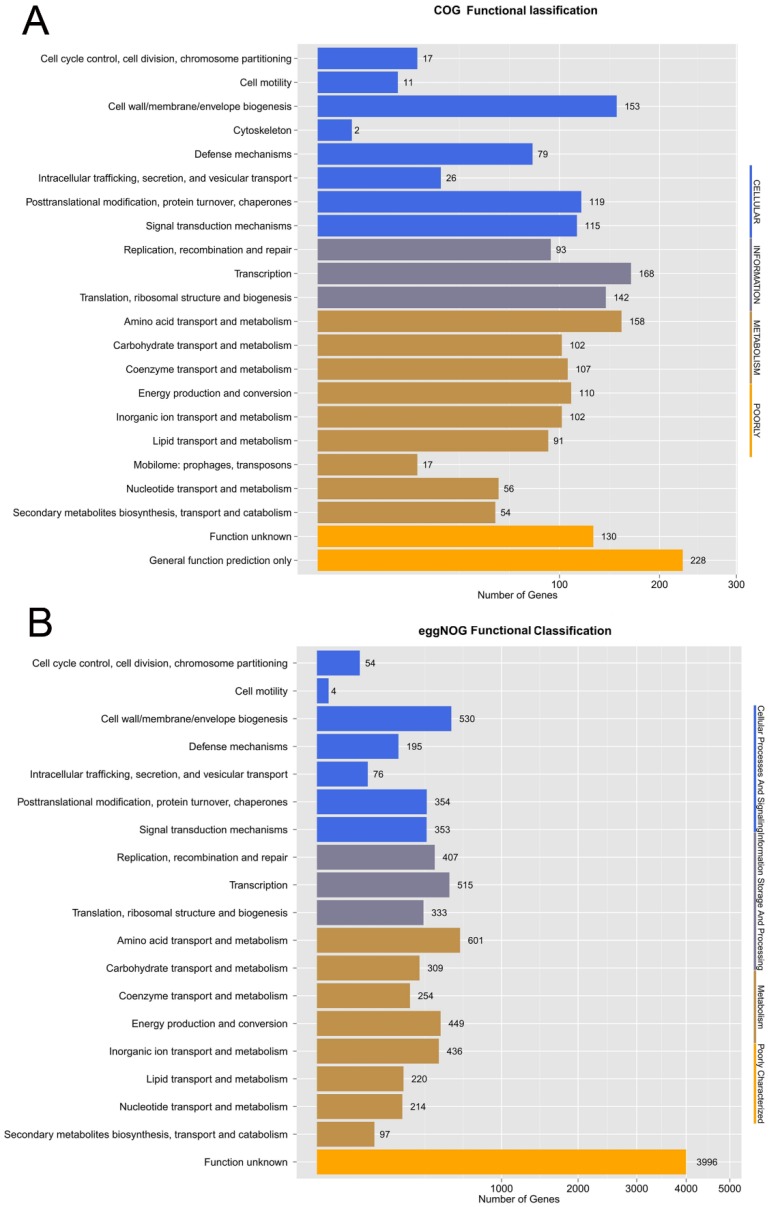

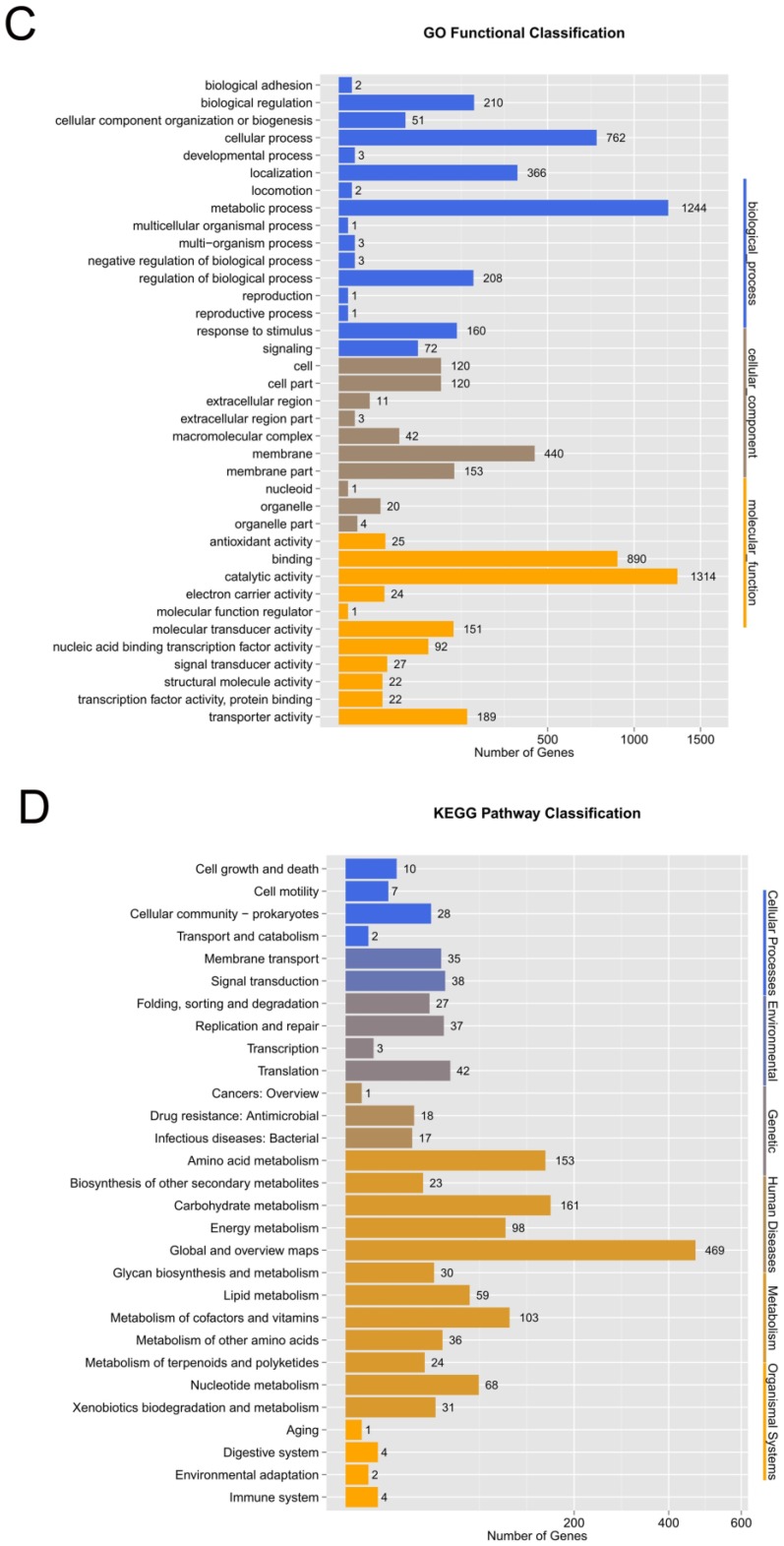
The functional annotations of *C. arthrosphaerae* ED882-96. (**A**) Cluster of orthologous gene (COG) classification. (**B**) eggNOG function classification. (**C**) Gene ontology (GO) functional classification. (**D**) Kyoto encyclopedia of genes and genomes (KEGG) pathway classification.

Orthologous genes are clusters of homologous genes that are descended from a single ancestral gene. Even after evolution, these genes usually retain functions similar to those of their ancestral genes [33]. We analyzed orthologous genes through the COG database, which is set in the NCBI. In the chromosomal genome of *C. arthrosphaerae* ED882-96, COGs related to “transcription”, “amino acid transport and metabolism”, and “cell wall/membrane/envelope biogenesis” were ranked as the largest parts. These COGs are essential for bacterial survival. Moreover, 79 COGs related to “defense mechanisms” were recognized in strain ED882-96 (Figure 4A).

eggNOG is a database of orthologous groups and function annotation of the genome. In contrast to the COG database, the eggNOG database is hosted by the European molecular biology laboratory. Although eggNOG is originated from COGs, this database is expanded to nonsupervised orthologous groups [19]. In the eggNOG function study of strain ED882-96, COGs associated with “amino acid transport and metabolism” accounted for the majority of COGs, followed by those associated with “cell wall/membrane/envelope and transcription”. The eggNOG analysis results revealed that the number of COGs associated with defense mechanisms was 195 (Figure 4B).

The genome of *C. arthrosphaerae* ED882-96 was then subjected to GO annotation classification and KEGG metabolic pathway analysis. GO is a bioinformatics database of formal ontologies that represent a comprehensively computational model of biological systems, including biological processes, molecular functions, and cellular components [20]. The GO analysis of strain ED882-96 showed that the most abundant subcategories in molecular functions were “catalytic activity”, followed by “metabolic process and binding” (Figure 4C). The KEGG database provides information of high-level functions for genome sequences and other high-throughput data regarding metabolism, genetic information processing, environmental information processing, cellular processes, organismal systems, human diseases, and drug development [22]. In the KEGG pathway classification, “global and overview maps”, “carbohydrate metabolism”, and “amino acid metabolism” were the first three largest parts of genes in strain ED882-96 (Figure 4D). The results of the KEGG pathway mapping of target gene candidates were similar to those of genome annotation performed using RAST.

### 3.6. Comparative Genomic Analysis of COGs and KEGG in Five C. arthrosphaerae Strains

**Figure 5:**
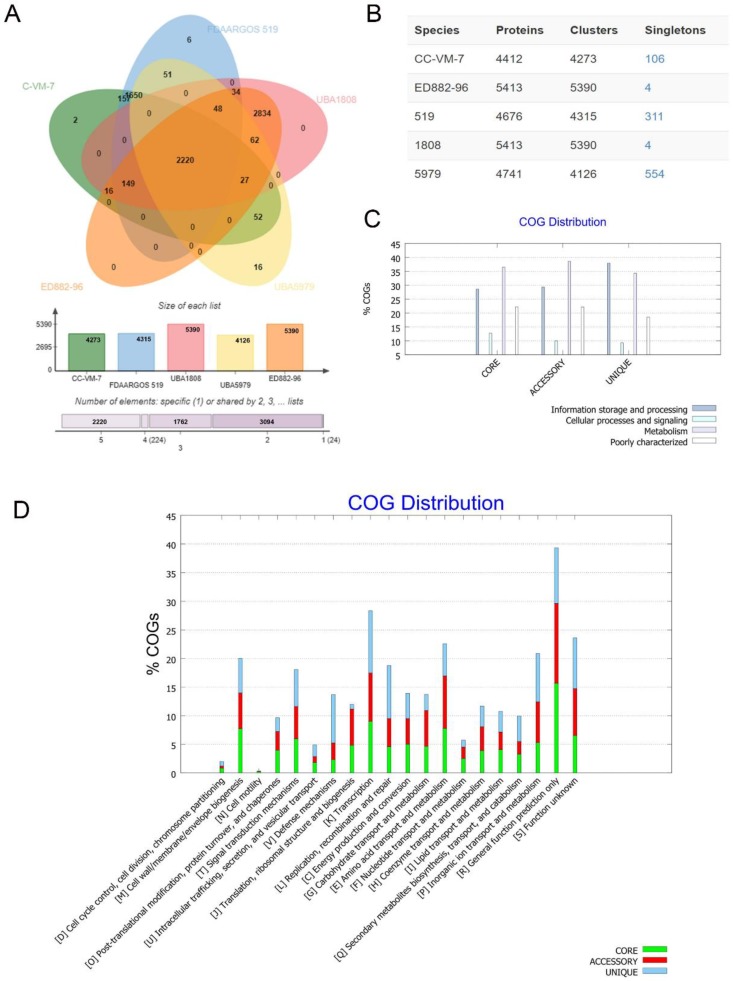
Proteome comparison among *C. arthrosphaerae* strains ED882-96, CC-VM-7^T^, FDAARGOS_519, UBA1808, and UBA5979. (**A**) The Venn diagram and bar chart represent the numbers of unique and shared orthologous genes of each strain. (**B**) The number of proteins, clusters, and singletons in each strain of *C. arthrosphaerae*. (**C**) Clusters of orthologous groups (COGs) in core, accessory, and unique genomes and their associated functions. (**D**) Detailed distribution of COGs with their functions.

The COGs of *C. arthrosphaerae* strain ED882-96 were compared with those of the other four *C. arthrosphaerae* strains available in the GenBank (Figure 5). A total of 2,200 COGs were shared by all five strains of *C. arthrosphaerae* (Figure 5A). The *C. arthrosphaerae* type strain CC-VM-7^T^ included 4273 COGs and 106 singletons. Strain ED882-96 comprised 5390 COGs and 4 singletons. Strain UBA5979 had the largest number of singletons (*n* = 554) (Figure 5B).

The functional analyses of COGs in the five strains of *C. arthrosphaerae* revealed that “metabolism”-related genes were the most abundant in the core (conserved) and accessory (dispensable) genomes. By contrast, COGs related to “information storage and processing” accounted for the most genes in the unique (strain-specific) genomes (Figure 5C).

Among the functional prediction of genomes, a majority of COGs were associated with R “general function prediction only” (Figure 5D). In the core genomes, 15.7%, 9%, and 7.8% of COGs had functions related to R, K “transcription”, and E “amino acid transport and metabolism”, respectively. In the accessory genomes, R accounted for 13.9% of COGs, E for 9.2%, and K for 8.4%. In the unique genomes, K was still the largest part of COGs (10.9%), followed by R (9.7%), and L “replication, recombination, and repair” (9.3%). Regarding COGs associated with antibiotic resistances, 2.3%, 2.9%, and 8.5% of core, accessory, and unique genomes, respectively, were related with V “defense mechanisms”. These results suggest that different strains of *C. arthrosphaerae* have developed diverse protective functions against external injury.

**Figure 6:**
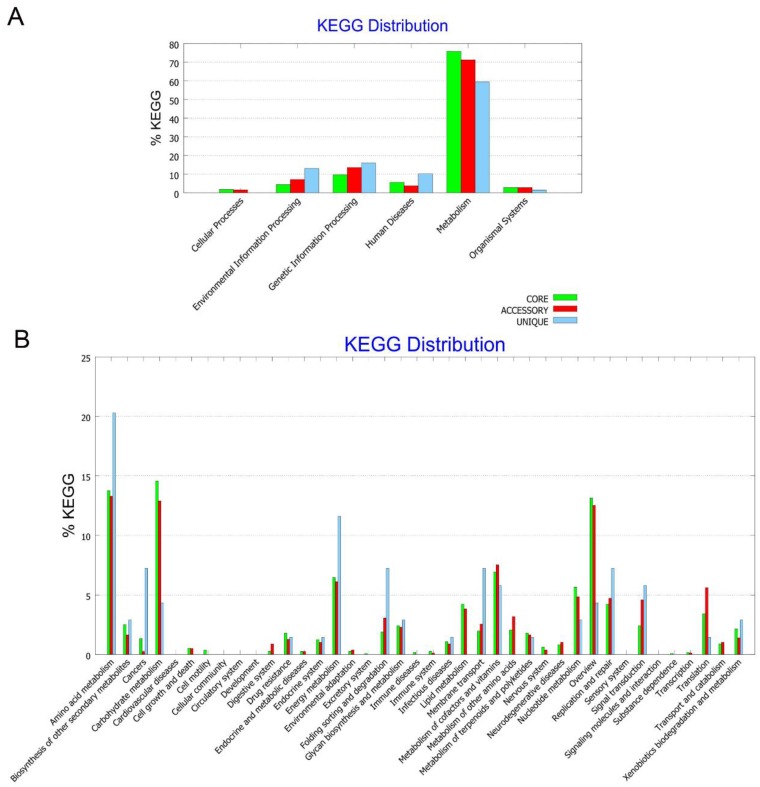
Kyoto encyclopedia of genes and genomes (KEGG) analysis of *C. arthrosphaerae* strain ED882-96, CC-VM-7^T^, FDAARGOS_519, UBA1808, and UBA5979. (**A**) KEGG pathway classification in core, accessory, and unique genomes. (**B**) Detailed distribution of KEGG pathway classification.

In the KEGG pathway analysis, genes associated with “metabolisms” accounted for the largest part in core, accessory, and unique genomes (Figure 6A). Of these genes, most of them were associated with “amino acid metabolism”, “carbohydrate metabolism”, “overview”, and “energy metabolism” (Figure 6B). Notably, 31 genes were associated with “drug resistances”, including 15 β-lactam resistance genes, five vancomycin resistance genes, and 11 cationic antimicrobial peptide resistance genes. Of these 31 genes, 20 were distributed in the core genome. These findings support that genes associated with antibiotic resistance play an essential role in these *C. arthrosphaerae* strains.

### 3.7. Virulence Factors

In this study, 83 virulence factor homologs were identified in *C. arthrosphaerae* ED882-96 using VFDB, including comprehensive products of capsule, lipopolysaccharide, hemolysin, endopeptidase, type VI secretion system, polyphosphate kinase, phenolic glycolipid biosynthesis and transport, macrophage infectivity potentiator, heat shock protein, catalase, two-component regulatory system, and others (Table S2). According to the functional classification scheme in VFDB, these virulence factors were related “offensive function”, “defensive function”, “nonspecific virulence factors”, and “regulation of virulence-associated gene” [23]. No study investigated the virulence of *C. arthrosphaerae* before. The findings of our study suggest that *C. arthrosphaerae* ED882-96 is a highly virulent strain. Further experiments, including animal studies, are necessary to verify these predicted virulent genes identified in this study.

### 3.8. Antimicrobial Resistance and Associated Genes

The MIC and susceptibility of *C. arthrosphaerae* strain ED882-96 are shown in Table 2. This strain was resistant to all tested antimicrobial agents, including β-lactams, β-lactam/β-lactamase inhibitor combinations, aminoglycosides, fluoroquinolones, tetracycline, glycylcycline, and trimethoprim-sulfamethoxazole.

No study has described the antimicrobial susceptibility patterns of *C. arthrosphaerae* before. Although other *Chryseobacterium* species usually express resistance to multiple antibiotics, these species are still susceptible to some antibiotics [27,34,35,36]. For example, previous studies have reported that 30–85% of *C. indologenes* were susceptible to piperacillin or piperacillin–tazobactam, 75% were susceptible to minocycline, 30–88% were susceptible to trimethoprim-sulfamethoxazole, and 32–85% were susceptible to fluoroquinolones [34,35,36]. However, our strain was resistant to all the antibiotics mentioned above.

Three major mechanisms of fluoroquinolone resistance are amino acid alteration in DNA gyrase (GyrA and GyrB) and topoisomerase IV (ParC and ParE), efflux pumps, and plasmid-mediated quinolone resistance (Qnr protein) [37]. *C. arthrosphaerae* ED882-96 was resistant to both ciprofloxacin and levofloxacin, and we examined whether mutations occurred in the QRDRs of the target genes. However, no nonsynonymous substitutions were observed in the QRDRs of GyrA, GyrB, ParC, or ParE. These results suggest that efflux pumps or Qnr protein could play roles in drug resistance to fluoroquinolones.

Antibiotic resistance genes were further examined using ARDB (Supplemental Table S3) and CARD (Supplemental Table S4). These antibiotic resistance genes include those with resistance to β-lactams, aminoglycosides, tetracyclines/glycylcycline, fluoroquinolones resistance, trimethoprim-sulfamethoxazole, macrolides, vancomycin, and chloramphenicol. In addition, a number of genes associated with multidrug efflux pumps were identified, such as multiple antibiotic resistance protein (MarA, MarB, MarC, MarR), tripartite multidrug resistance system, multidrug and toxin extrusion family efflux pump (YdhE/NorM), major facilitator superfamily, multidrug efflux pump component, resistance-nodulation-division (RND) efflux system (CmeA, CmeB, amd CmeC), multidrug efflux RND transporter (MexD), MexD membrane fusion protein (MexC), and multidrug resistance efflux pump (PmrA). The findings of many antibiotic resistance genes in the genome *C. arthrosphaerae* ED882-96 are consistent with the fact that this strain exhibits extreme resistance to all tested antibiotics.

## 4. Conclusions

The present study describes the genomic characteristics, antimicrobial susceptibility pattern, and comparative genomics of *C. arthrosphaerae* strain ED882-96 isolated from the blood of a patient in Taiwan. Our study suggests that *C. arthrosphaerae* is a highly virulent and multidrug-resistant strain. The results of this study provide crucial knowledge to understand the phylogenetic distinctness, genomic features, and antibiotic susceptibility pattern of this uncommon *Chryseobacterium* species.

## Figures and Tables

**Table 1:** Assembly and annotation statistics.

Assembly and annotation	Chromosome	Plasmid
Total sequence length (bp)	4,249,864	435,667
Number of contigs	2	1
Contig N50 (bp)	2,386,100	435,667
Contig L50	1	1
GC content (%)	38.3	38.1
Number of subsystems	191	29
Number of coding sequences	5413	512
Number of rRNA	6	3
Number of tRNA	37	22

**Table 2:** The minimum inhibitory concentration and susceptibility of *C. arthrosphaerae* ED882-96.

Antibiotic Group	Antibiotics	MIC	Interpretation
β-lactams, β-lactam/β-lactamase inhibitors	Piperacillin	>64	R
	Piperacillin-tazobactam	>128/4	R
	Ticarcillin-clavulanic acid	>64/2	R
	Ceftazidime	>16	R
	Cefepime	>32	R
	Ceftriaxone	>32	R
	Aztreonam	>16	R
	Imipenem	>8	R
	Meropenem	>8	R
Aminoglycosides	Gentamicin	>8	R
	Tobramycin	>8	R
	Amikacin	>32	R
Tetracyclines/glycylcycline	Tetracycline	>8	R
	Minocycline	>8	R
	Tigecycline	>8	R
Fluoroquinolones	Ciprofloxacin	>2	R
	Levofloxacin	>8	R
Folate pathway inhibitors	Trimethoprim-sulfamethoxazole	>4/76	R

MIC, minimum inhibitory concentration.

## Supplementary Materials

The following are available online at https://www.mdpi.com/2073-4425/10/4/309/s1, Table S1: Primers and protocols for amplification and sequencing conditions used in this study, Table S2: Virulence factors predicted using Virulence Factors Database (VFDB), Table S3: Genes associated with antibiotic resistance predicted using Antibiotic Resistance Genes Database (ARDB), Table S4: Genes associated with antibiotic resistance predicted using Comprehensive Antibiotic Resistance Database (CARD).

## References

[1] Vandamme P., Bernardet J.F., Segers P., Kersters K., Holmes B. (1994). New perspectives in the classification of the *Flavobacteria*: Description of *Chryseobacterium* gen. nov., *Bergeyella* gen. nov., and *Empedobacter* nom. rev. Int. J. Syst. Evol. Microbiol..

[2] Kämpfer P., Arun A.B., Young C.C., Chen W.M., Sridhar K.R., Rekha P.D. (2010). *Chryseobacterium arthrosphaerae* sp. nov., isolated from the faeces of the pill millipede *Arthrosphaera magna* Attems. Int. J. Syst. Evol. Microbiol..

[3] Jeong J.J., Lee D.W., Park B., Sang M.K., Choi I.G., Kim K.D. (2017). *Chryseobacterium cucumeris* sp. nov., an endophyte isolated from cucumber (*Cucumis sativus* L.) root, and emended description of *Chryseobacterium arthrosphaerae*. Int. J. Syst. Evol. Microbiol..

[4] Chryseobacterium. http://www.bacterio.net/chryseobacterium.html.

[5] Lin J.N., Lai C.H., Yang C.H., Huang Y.H., Lin H.F., Lin H.H. (2017). Comparison of four automated microbiology systems with 16S rRNA gene sequencing for identification of *Chryseobacterium* and *Elizabethkingia* species. Sci. Rep..

[6] Lin J.N., Teng S.H., Lai C.H., Yang C.H., Huang Y.H., Lin H.F., Lin H.H. (2018). Comparison of the Vitek MS and Bruker matrix-assisted laser desorption ionization-time of flight mass spectrometry systems for identification of *Chryseobacterium* isolated from clinical specimens and report of uncommon *Chryseobacterium* infections in humans. J. Clin. Microbiol..

[7] Kumar S., Stecher G., Tamura K. (2016). MEGA7: Molecular evolutionary genetics analysis version 7.0 for bigger datasets. Mol. Biol. Evol..

[8] Letunic I., Bork P. (2019). Interactive Tree Of Life (iTOL) v4: Recent updates and new developments. Nucleic Acids Res..

[9] Hurgobin B. (2016). Short read alignment using SOAP2. Methods Mol. Biol. Clifton NJ.

[10] Chin C.S., Alexander D.H., Marks P., Klammer A.A., Drake J., Heiner C., Clum A., Copeland A., Huddleston J., Eichler E.E. (2013). Non-hybrid, finished microbial genome assemblies from long-read SMRT sequencing data. Nat. Methods.

[11] Li S., Li R., Li H., Lu J., Li Y., Bolund L., Schierup M.H., Wang J. (2013). SOAPindel: Efficient identification of indels from short paired reads. Genome Res..

[12] Lee I., Kim Y.O., Park S.C., Chun J. (2015). OrthoANI: An improved algorithm and software for calculating average nucleotide identity. Int. J. Syst. Evol. Microbiol..

[13] Richter M., Rosselló-Móra R. (2009). Shifting the genomic gold standard for the prokaryotic species definition. Proc. Natl. Acad. Sci. USA.

[14] Meier-Kolthoff J.P., Auch A.F., Klenk H.P., Göker M. (2013). Genome sequence-based species delimitation with confidence intervals and improved distance functions. BMC Bioinform..

[15] Tatusova T., DiCuccio M., Badretdin A., Chetvernin V., Nawrocki E.P., Zaslavsky L., Lomsadze A., Pruitt K.D., Borodovsky M., Ostell J. (2016). NCBI prokaryotic genome annotation pipeline. Nucleic Acids Res..

[16] Aziz R.K., Bartels D., Best A.A., DeJongh M., Disz T., Edwards R.A., Formsma K., Gerdes S., Glass E.M., Kubal M. (2008). The RAST Server: Rapid annotations using subsystems technology. BMC Genom..

[17] Overbeek R., Olson R., Pusch G.D., Olsen G.J., Davis J.J., Disz T., Edwards R.A., Gerdes S., Parrello B., Shukla M. (2014). The SEED and the rapid annotation of microbial genomes using subsystems technology (RAST). Nucleic Acids Res..

[18] Galperin M.Y., Makarova K.S., Wolf Y.I., Koonin E.V. (2015). Expanded microbial genome coverage and improved protein family annotation in the COG database. Nucleic Acids Res..

[19] Huerta-Cepas J., Szklarczyk D., Forslund K., Cook H., Heller D., Walter M.C., Rattei T., Mende D.R., Sunagawa S., Kuhn M. (2016). eggNOG 4.5: A hierarchical orthology framework with improved functional annotations for eukaryotic, prokaryotic and viral sequences. Nucleic Acids Res..

[20] Gene Ontology Consortium (2008). The gene ontology project in 2008. Nucleic Acids Res..

[21] Jones P., Binns D., Chang H.-Y., Fraser M., Li W., McAnulla C., McWilliam H., Maslen J., Mitchell A., Nuka G. (2014). InterProScan 5: Genome-scale protein function classification. Bioinformatics.

[22] Kanehisa M., Sato Y., Furumichi M., Morishima K., Tanabe M. (2018). New approach for understanding genome variations in KEGG. Nucleic Acids Res..

[23] Chen L., Zheng D., Liu B., Yang J., Jin Q. (2016). VFDB 2016: Hierarchical and refined dataset for big data analysis—10 years on. Nucleic Acids Res..

[24] Grant J.R., Stothard P. (2008). The CGView Server: A comparative genomics tool for circular genomes. Nucleic Acids Res..

[25] Clinical and Laboratory Standards Institute (2017). Performance Standards for Antimicrobial Susceptibility Testing.

[26] Kelesidis T., Karageorgopoulos D.E., Kelesidis I., Falagas M.E. (2008). Tigecycline for the treatment of multidrug-resistant *Enterobacteriaceae*: A systematic review of the evidence from microbiological and clinical studies. J. Antimicrob. Chemother..

[27] Lin J.N., Lai C.H., Yang C.H., Huang Y.H. (2019). Differences in clinical manifestations, antimicrobial susceptibility patterns and mutations of fluoroquinolone target genes between *Chryseobacterium gleum* and *Chryseobacterium indologenes*. Antimicrob. Agents Chemother..

[28] Liu B., Pop M. (2009). ARDB—antibiotic resistance genes database. Nucleic Acids Res..

[29] Jia B., Raphenya A.R., Alcock B., Waglechner N., Guo P., Tsang K.K., Lago B.A., Dave B.M., Pereira S., Sharma A.N. (2017). CARD 2017: Expansion and model-centric curation of the comprehensive antibiotic resistance database. Nucleic Acids Res..

[30] Wang Y., Coleman-Derr D., Chen G., Gu Y.Q. (2015). OrthoVenn: A web server for genome wide comparison and annotation of orthologous clusters across multiple species. Nucleic Acids Res..

[31] Chaudhari N.M., Gupta V.K., Dutta C. (2016). BPGA—An ultra-fast pan-genome analysis pipeline. Sci. Rep..

[32] How K.Y., Hong K.W., Sam C.K., Koh C.L., Yin W.F., Chan K.G. (2015). Unravelling the genome of long chain N-acylhomoserine lactone-producing *Acinetobacter* sp. strain GG2 and identification of its quorum sensing synthase gene. Front. Microbiol..

[33] Tatusov R.L., Fedorova N.D., Jackson J.D., Jacobs A.R., Kiryutin B., Koonin E.V., Krylov D.M., Mazumder R., Mekhedov S.L., Nikolskaya A.N. (2003). The COG database: An updated version includes eukaryotes. BMC Bioinform..

[34] Hsueh P.R., Teng L.J., Yang P.C., Ho S.W., Hsieh W.C., Luh K.T. (1997). Increasing incidence of nosocomial *Chryseobacterium indologenes* infections in Taiwan. Eur. J. Clin. Microbiol..

[35] Kirby J.T., Sader H.S., Walsh T.R., Jones R.N. (2004). Antimicrobial susceptibility and epidemiology of a worldwide collection of *Chryseobacterium* spp: Report from the SENTRY antimicrobial surveillance program (1997–2001). J. Clin. Microbiol..

[36] Chen F.L., Wang G.C., Teng S.O., Ou T.Y., Yu F.L., Lee W.S. (2013). Clinical and epidemiological features of *Chryseobacterium indologenes* infections: Analysis of 215 cases. J. Microbiol. Immunol. Infect..

[37] Jacoby G.A. (2005). Mechanisms of resistance to quinolones. Clin. Infect. Dis..

