# Supplementary material for: Genomic Features, Comparative Genomic Analysis, and Antimicrobial Susceptibility Patterns of Chryseobacterium arthrosphaerae Strain ED882-96 Isolated in Taiwan
Source: Genes (Basel). 2019 Apr 20;10(4):309. doi: 10.3390/genes10040309 (PMC6523182; doi:10.3390/genes10040309)
Supplement: Supplementary file 1 [file genes-10-00309-s001.pdf]

Genomic Features, Comparative Genomic Analysis, and Antimicrobial Susceptibility Patters of *Chryseobacterium arthrosphaerae* Strain ED882-96 Isolated in Taiwan

Chih-Yu Liang, Chih-Hui Yang, Chung-Hsu Lai, Yi-Han Huang and Jiun-Nong Lin

Table S1. Primers and protocols for amplification and sequencing conditions used in this study.

Primer	Sequence (5' to 3')	Amplicon Size (bp)	GenBank Accession No. or Reference
PCR of 16S rRNA			
8f	CACGGATCCAGACTTTGAT(C/T)(A/C)TGGCTCAG	1498	Reference 1
1512r	GTGAAGCTTACGG(C/T)TAGCTTGTTACGACTT		
Sequencing of 16S rRNA			
8f	CACGGATCCAGACTTTGAT(C/T)(A/C)TGGCTCAG		
534r	ATTACCGCGGCTGCTGG		
534f	CCAGCAGCCGCGGTAAT		Reference 1<br>This study
968f	AACGCGAAGAACCTTAC		
1512r	GTGAAGCTTACGG(C/T)TAGCTTGTTACGACTT		
PCR and sequencing of QRDR for *C. gleum*			
gyrA-f[a]	TCGGTTATCGTTTCCAGAGC	466	
gyrA-r[a]	TGTTGGTTGCCATCCCTACT		
gyrB-f[b]	CTCTGAAGTTTCAGGGGCTGT		This study
gyrB-r[b]	CAATATCGGCATCGGTCATG	491	Accession no. GL379781
parC-f[b]	CTGGTTYCTKGATTATGCTTCC		
parC-r[b]	GCTCTTACTTTTCCTCCTCTGTG	609	

parE-f[b]	GCATTCCGTGAAGCATATG	997
parE-r[b]	TTTGTCTGTCCGGAGTGTTTT	

[a]The conditions were as follows: an initial extended denaturation step of 94°C for 5 min, followed by 30 cycles of 30 s at 94°C, 30 s at 55°C, 1 min at 72°C, and a final 5 min at 72°C.

[b]The conditions were as follow: an initial extended denaturation step of 94°C for 5 min, followed by 32 cycles of 30 s at 94°C, 30 s at 50°C, 1 min at 72°C, and a final 5 min at 72°C.

Table S2. Virulence factors predicted using Virulence Factors Database (VFDB).

No.	Gene Id	Identity	E value	Description
1	88296-15GL001440	41.53	7.00E-20	(pks15) polyketide synthase PKS15 [PDIM (phthiocerol dimycocerosate) and PGL (phenolic glycolipid) biosynthesis and transport (CVF288)] [Mycobacterium tuberculosis RGTB423]
2	88296-15GL001565	53.23	5.00E-32	(ureG) urease/hydrogenase-associated predicted GTPase UreG [Urease (CVF221)] [Helicobacter hepaticus ATCC 51449]
3	88296-15GL002309	68.72	3.00E-150	(CT396) molecular chaperone DnaK [MOMP (AI392)] [Chlamydia trachomatis D/UW-3/CX]
4	88296-15GL002330	40.88	8.00E-63	(ppk) polyphosphate kinase [type IV pili (AI117)] [Dichelobacter nodosus VCS1703A]
5	88296-15GL002458	41.56	1.00E-28	(rtxB) RtxB protein [RTX toxin (CVF263)] [Vibrio cholerae O395]
6	88296-15GL002477	48.03	4.00E-74	(hemB) Porphobilinogen synthase [Heme biosynthesis (CVF506)] [Haemophilus somnus 2336]
7	88296-15GL002498	51.52	1.00E-14	(fabZ) (3R)-hydroxymyristoyl ACP dehydratase [LPS (CVF383)] [Brucella suis 1330]
8	88296-15GL002527	55.74	2.00E-115	(capL) hypothetical protein [Capsule (CVF110)] [Staphylococcus haemolyticus JCSC1435]
9	88296-15GL002535	46.58	1.00E-65	(capK) hypothetical protein [Capsule (CVF110)] [Staphylococcus haemolyticus JCSC1435]

10	88296-15GL002537	66.67	3.00E-84	(ABTJ_03752) UDP-N-acetylglucosamine 2-epimerase [Capsule (CVF775)] [Acinetobacter baumannii MDR-TJ]
11	88296-15GL002542	68.73	2.00E-135	(fnlA) UDP-N-acetylglucosamine 4,6-dehydratase [Capsule (CVF775)] [Acinetobacter baumannii D1279779]
12	88296-15GL002544	49.17	3.00E-30	(PSPA7_1979) imidazole glycerol phosphate synthase subunit HisF [LPS O-antigen (P. aeruginosa) (CVF520)] [Pseudomonas aeruginosa PA7]
13	88296-15GL002545	44.12	6.00E-49	(hisH2) glutamine amidotransferase [LPS O-antigen (P. aeruginosa) (CVF520)] [Pseudomonas aeruginosa PAO1]
14	88296-15GL002558	41.08	4.00E-80	(wbpG) LPS biosynthesis protein WbpG [LPS O-antigen (P. aeruginosa) (CVF520)] [Pseudomonas aeruginosa PAO1]
15	88296-15GL002559	52.24	3.00E-73	(hisF2) imidazole glycerol phosphate synthase subunit HisF [LPS O-antigen (P. aeruginosa) (CVF520)] [Pseudomonas aeruginosa PAO1]
16	88296-15GL002560	50.74	1.00E-59	(hisH2) glutamine amidotransferase [LPS O-antigen (P. aeruginosa) (CVF520)] [Pseudomonas aeruginosa PAO1]
17	88296-15GL002561	53.28	3.00E-26	(A1S_0057) capsular polysaccharide synthesis enzyme [Capsule (CVF775)] [Acinetobacter baumannii ATCC 17978]
18	88296-15GL002565	53.33	1.00E-63	(wbpI) probable UDP-N-acetylglucosamine 2-epimerase WbpI [LPS O-antigen (P. aeruginosa) (CVF520)] [Pseudomonas aeruginosa PAO1]
19	88296-15GL002568	55.06	3.00E-47	(wbpD) probable acetyltransferase WbpD [LPS O-antigen (P. aeruginosa) (CVF520)] [Pseudomonas aeruginosa PAO1]
20	88296-15GL002571	54.04	2.00E-46	(wbpE) probable aminotransferase WbpE [LPS O-antigen (P. aeruginosa) (CVF520)] [Pseudomonas aeruginosa PAO1]
21	88296-15GL002573	44.87	1.00E-64	(ipaH) hypothetical protein [Mxi-Spa TTSS effectors controlled by MxiE (CVF465)] [Shigella flexneri 2a str. 2457T]
22	88296-15GL002574	56.58	3.00E-144	(PLES_19091) UDP-glucose/GDP-mannose dehydrogenase-like protein [LPS O-antigen (P. aeruginosa) (CVF520)] [Pseudomonas aeruginosa LESB58]
23	88296-15GL002579	43.94	7.00E-75	(BCE_5395) caspsular polysaccharide biosynthesis protein [Polysaccharide capsule

				(CVF567)] [Bacillus cereus ATCC 10987]
24	88296-15GL002593	41.08	6.00E-31	(aatC) ABC transporter ATP-binding protein AatC [ABC transporter for dispersin (CVF737)] [Escherichia coli O78:H11:K80 str. H10407]
25	88296-15GL002711	53.15	1.00E-29	(panD) aspartate 1-decarboxylase [Pantothenate synthesis (CVF305)] [Mycobacterium abscessus subsp. bolletii str. GO 06]
26	88296-15GL002736	43.39	5.00E-65	(pdhB) pyruvate dehydrogenase E1 component beta subunit [PDH-B (CVF588)] [Mycoplasma mobile 163K]
27	88296-15GL002794	59.79	6.00E-68	(icl) isocitrate lyase [Isocitrate lyase (CVF302)] [Mycobacterium sp. KMS]
28	88296-15GL002795	60.19	1.00E-73	(icl) isocitrate lyase [Isocitrate lyase (CVF302)] [Mycobacterium avium 104]
29	88296-15GL002831	50	1.00E-35	(mgtC) hypothetical protein [Mg2+ transport (CVF005)] [Salmonella enterica subsp. arizonae serovar 62:z4,z23:-- str. RSK2980]
30	88296-15GL002894	45.16	9.00E-24	(ricA) Rab2 interacting conserved protein A [RicA (VF0414)] [Brucella melitensis bv. 1 str. 16M]
31	88296-15GL002919	44.31	2.00E-104	(adeG) RND family efflux transporter [AdeFGH efflux pump/transport autoinducer (CVF773)] [Acinetobacter baumannii AB0057]
32	88296-15GL003039	49.4	2.00E-87	(MG_301) glyceraldehyde-3-phosphate dehydrogenase [GAPDH (AI368)] [Mycoplasma genitalium G37]
33	88296-15GL003151	66.1	0	(htpB) Hsp60, 60K catalase HtpB [Hsp60 (CVF347)] [Legionella pneumophila str. Corby]
34	88296-15GL003239	41.44	8.00E-71	(msbA) lipid A export ATP-binding protein MsbA [LOS (CVF494)] [Haemophilus influenzae PittGG]
35	88296-15GL003318	67.18	5.00E-165	(tuf) translation elongation factor Tu [EF-Tu (CVF587)] [Mycoplasma mycoides subsp. mycoides SC str. PG1]
36	88296-15GL003400	58.82	5.00E-10	(acpXL) acyl carrier protein [LPS (CVF383)] [Brucella melitensis bv. 1 str. 16M]
37	88296-15GL003401	40.94	1.00E-62	(kasB) 3-oxoacyl-(acyl carrier protein) synthase II [FAS-II (CVF300)] [Mycobacterium tuberculosis RGTB327]
38	88296-15GL003497	41.45	1.00E-30	(BCE_5392) glycosyl transferase, group 2 family protein [Polysaccharide capsule

				(CVF567)] [Bacillus cereus ATCC 10987]
39	88296-15GL003553	40.83	2.00E-19	(C8J_1075) hypothetical protein [LOS (CVF396)] [Campylobacter jejuni subsp. jejuni 81116]
40	88296-15GL003630	40.18	1.00E-34	(piplc) 1-phosphatidylinositol phosphodiesterase [Phosphatidylinositol-specific phospholipase C (PI-PLC) (CVF570)] [Bacillus cereus ATCC 10987]
41	88296-15GL003672	44.29	2.00E-49	(rmlD) dTDP-4-dehydrorhamnose reductase [Capsular polysaccharide (CVF282)] [Vibrio vulnificus YJ016]
42	88296-15GL003678	43.53	2.00E-53	(cpsA) undecaprenyl diphosphate synthase [Capsule (VF0361)] [Enterococcus faecalis V583]
43	88296-15GL003680	44.23	4.00E-14	(BCE_5400) capsular exopolysaccharide family protein [Polysaccharide capsule (CVF567)] [Bacillus cereus ATCC 10987]
44	88296-15GL003689	46.62	3.00E-31	(BC5267) Glycosyltransferase [Polysaccharide capsule (CVF567)] [Bacillus cereus ATCC 14579]
45	88296-15GL003716	40.8	1.00E-31	(ddrA) daunorubicin resistance ABC transporter ATPase subunit [PDIM (phthiocerol dimycocerosate) and PGL (phenolic glycolipid) biosynthesis and transport (CVF288)] [Mycobacterium vanbaalenii PYR-1]
46	88296-15GL003753	41.53	2.00E-29	(kasB) 3-oxoacyl-(acyl carrier protein) synthase II [FAS-II (CVF300)] [Mycobacterium sp. JDM601]
47	88296-15GL003754	42.62	1.00E-50	(flmH) 3-oxoacyl-ACP reductase [Polar flagella (VF0473)] [Aeromonas hydrophila ML09-119]
48	88296-15GL003790	52.46	1.00E-140	(katA) catalase [Catalase (CVF760)] [Neisseria lactamica 020-06]
49	88296-15GL003838	44.92	7.00E-54	(qbsC) QbsC [thioquinolobactin (IA009)] [Pseudomonas fluorescens ATCC 17400]
50	88296-15GL003913	64.9	6.00E-163	(pgi) glucose-6-phosphate isomerase [Exopolysaccharide (CVF495)] [Haemophilus influenzae 86-028NP]
51	88296-15GL003918	43.84	2.00E-26	(BCE_5393) UDP-galactose phosphate transferase [Polysaccharide capsule (CVF567)] [Bacillus cereus ATCC 10987]
52	88296-15GL004095	50.64	3.00E-38	(BCE_5384) NAD dependent epimerase/dehydratase family protein [Polysaccharide

				capsule (CVF567)] [Bacillus cereus ATCC 10987]
53	88296-15GL004177	42.59	9.00E-23	(lisR) two-component response regulator [LisR/LisK (CVF253)] [Listeria monocytogenes EGD-e]
54	88296-15GL004210	40.86	2.00E-55	(hlyB) hemolysin transport protein [Hemolysin (VF0207)] [Escherichia coli O157:H7 str. EDL933]
55	88296-15GL004213	45.35	3.00E-17	(irtA) drugs-transport transmembrane ATP-binding protein ABC transporter [ABC transporter (CVF316)] [Mycobacterium tuberculosis CAS/NITR204]
56	88296-15GL004342	51.46	6.00E-63	(flmH) 3-oxoacyl-ACP reductase [Polar flagella (VF0473)] [Aeromonas hydrophila ML09-119]
57	88296-15GL004395	53.92	3.00E-101	(clpC) endopeptidase Clp ATP-binding chain C [ClpC (VF0072)] [Listeria monocytogenes EGD-e]
58	88296-15GL005043	50.64	4.00E-63	(hemL) glutamate-1-semialdehyde aminotransferase [Heme biosynthesis (CVF506)] [Haemophilus somnus 129PT]
59	88296-15GL005449	41.42	3.00E-24	(farA) putative efflux pump protein [FarAB (CVF758)] [Neisseria meningitidis alpha14]
60	88296-15GL005513	58.82	1.00E-38	(ureA) urease alpha subunit [Urease (CVF221)] [Helicobacter acinonychis str. Sheeba]
61	88296-15GL005514	71.25	1.00E-129	(ureB) urease [Urease (CVF221)] [Helicobacter hepaticus ATCC 51449]
62	88296-15GL005520	51.43	3.00E-12	(ureE) urease accessory protein UreE [Urease (CVF221)] [Helicobacter pylori Shi470]
63	88296-15GL005738	58.23	4.00E-73	(IlpA) immunogenic lipoprotein A [IlpA (VF0513)] [Vibrio vulnificus YJ016]
64	88296-15GL006115	70.41	4.00E-29	(evpJ) type VI secretion system protein EvpJ [EVP (E. tarda virulent protein) (SS189)] [Edwardsiella tarda EIB202]
65	88296-15GL006741	42.36	3.00E-62	(clbD) putative 3-hydroxyacyl-CoA dehydrogenase [colibactin (TX033)] [Escherichia coli O18:K1:H7 str. IHE3034]
66	88296-15GL007198	44.68	2.00E-13	(cap8J) capsular polysaccharide synthesis enzyme [Capsule (CVF110)] [Staphylococcus aureus subsp. aureus MRSA252]

67	88296-15GL007227	55.73	1.00E-40	(kpsF) arabinose-5-phosphate isomerase [LOS (CVF494)] [Haemophilus ducreyi 35000HP]
68	88296-15GL007406	44.62	1.00E-51	(BCAM2307) zinc metalloprotease ZmpB [ZmpB (TX176)] [Burkholderia cenocepacia J2315]
69	88296-15GL007555	42.21	1.00E-59	(kdsA) 2-dehydro-3-deoxyphosphooctonate aldolase [LOS (CVF494)] [Haemophilus somnus 129PT]
70	88296-15GL007576	42.91	2.00E-53	(rpoS) RNA polymerase sigma factor RpoS [Alternative sigma factor RpoS (CVF364)] [Legionella longbeachae NSW150]
71	88296-15GL007764	50.9	1.00E-94	(dfoJ) putative decarboxylase involved in desferrioxamine biosynthesis [desferrioxamine (IA033)] [Erwinia amylovora CFBP1430]
72	88296-15GL007770	40.19	4.00E-64	(dfoA) L-lysine 6-monooxygenase involved in desferrioxamine biosynthesis [desferrioxamine (IA033)] [Erwinia amylovora CFBP1430]
73	88296-15GL007919	41.98	1.00E-69	(lpg0041) Dot/Icm type IV secretion system effector [Dot/Icm (VF0156)] [Legionella pneumophila subsp. pneumophila str. Philadelphia 1]
74	88296-15GL008018	46.67	1.00E-42	(hisF) imidazole glycerol phosphate synthase subunit HisF [LPS (VF0171)] [Legionella pneumophila subsp. pneumophila str. Philadelphia 1]
75	88296-15GL008021	41.58	2.00E-16	(hisH2) glutamine amidotransferase [LPS O-antigen (P. aeruginosa) (CVF520)] [Pseudomonas aeruginosa PAO1]
76	88296-15GL008105	41.24	2.00E-94	(adeG) RND family efflux transporter [AdeFGH efflux pump/transport autoinducer (CVF773)] [Acinetobacter baumannii AB0057]
77	88296-15GL008325	52.1	9.00E-69	(pilR) putative two-component system, response regulator [Type IV pili biosynthesis (CVF518)] [Pseudomonas fluorescens SBW25]
78	88296-15GL008449	47.53	2.00E-39	(lirB) Dot/Icm type IV secretion system effector LirB [Dot/Icm (SS047)] [Legionella pneumophila subsp. pneumophila str. Philadelphia 1]
79	88296-15GL008469	48.57	8.00E-11	(ndk) nucleoside diphosphate kinase [Nucleoside diphosphate kinase (CVF660)] [Mycobacterium smegmatis JS623]
80	88296-15GL008563	43.62	8.00E-28	(kdtB) lipopolysaccharide core biosynthesis protein [LPS (VF0056)] [Helicobacter

				pylori 26695]
81	88296-15GL008797	48.25	1.00E-35	(mip) macrophage infectivity potentiator [Mip (CVF349)] [Legionella longbeachae NSW150]
82	88296-15GL008872	50.29	3.00E-41	(panC) pantoate--beta-alanine ligase [Pantothenate synthesis (CVF305)] [Mycobacterium smegmatis str. MC2 155]
83	88296-15GL009193	43.64	9.00E-22	(mgtC) hypothetical protein [Mg2+ transport (CVF005)] [Salmonella enterica subsp. arizonae serovar 62:z4,z23:-- str. RSK2980]

Table S3. Genes associated with antibiotic resistance predicted using Antibiotic Resistance Genes Database (ARDB).

No.	Gene Id	Identity	E value	Resistance Type	Antibiotic Resistance	Description
1	88296-15GL002550	45.92	4.00E-17	catb3	chloramphenicol	Group B chloramphenicol acetyltransferase, which can inactivate chloramphenicol. Also referred to as xenobiotic acetyltransferase.
2	88296-15GL002919	44.33	2.00E-121	acrb	aminoglycoside, glycylcycline, macrolide, beta-lactam, acriflavin	Resistance-nodulation-cell division transporter system. Multidrug resistance efflux pump.
3	88296-15GL002920	40.24	3.00E-106	ceob	chloramphenicol	Resistance-nodulation-cell division transporter system. Multidrug resistance efflux pump.
4	88296-15GL003399	75	1.00E-62	catb3	chloramphenicol	Group B chloramphenicol acetyltransferase, which can inactivate chloramphenicol. Also referred to as xenobiotic acetyltransferase.
5	88296-15GL003716	41.57	9.00E-25	bcra	bacitracin	ABC transporter system, bacitracin efflux pump.
6	88296-15GL004177	41.54	7.00E-26	vanre	vancomycin	VanE type vancomycin resistance operon genes, which can synthesize peptidoglycan with modified C-terminal D-Ala-D-Ala to D-alanine--D-serine.

7	88296-15GL006031	46.6	5.00E-42	bl2_veb	cephalosporin, penicillin	Class A beta-lactamase. This enzyme breaks the beta-lactam antibiotic ring open and deactivates the molecule's antibacterial properties.
8	88296-15GL006890	52.29	1.00E-26	emre	aminoglycoside	Multidrug resistance efflux pump.
9	88296-15GL007471	51.8	2.00E-33	bl2_veb	cephalosporin, penicillin	Class A beta-lactamase. This enzyme breaks the beta-lactam antibiotic ring open and deactivates the molecule's antibacterial properties.
10	88296-15GL008105	42.38	4.00E-104	mexf	chloramphenicol, fluoroquinolone	Resistance-nodulation-cell division transporter system. Multidrug resistance efflux pump.
11	88296-15GL008343	43.52	9.00E-16	ykkd	na_antimicrobials	Small Multidrug Resistance (SMR) protein family. Multidrug resistance efflux pump, which consists of two proteins.
12	88296-15GL008565	43.64	1.00E-28	vang	vancomycin	VanG type vancomycin resistance operon genes, which can synthesize peptidoglycan with modified C-terminal D-Ala-D-Ala to D-alanine--D-serine.

Table S4. Genes associated with antibiotic resistance predicted using Comprehensive Antibiotic Resistance Database (CARD).

No.	Gene Id	Identity	E value	Classification	Definition of Term
1	88296-15GL002035	63.26	1.00E-102	antibiotic target|mechanism of antibiotic resistance|determinant of antibiotic resistance|antibiotic molecule	Point mutations in the thymidylate synthetase thyA gene shown clinically to confer resistance to para-aminosalicylic acid. Loss-of-function mutations in thyA disrupt the catalytic activity and substrate-binding affinity, thus conferring resistance. [PMID:25421465]
2	88296-15GL002550	45.92	3.00E-17	mechanism of antibiotic resistance|determinant of antibiotic resistance|antibiotic molecule	catB8 is a plasmid or integron-encoded variant of the cat gene found in Klebsiella pneumoniae, Salmonella typhi and Pseudomonas aeruginosa [PMID:12760886]

3	88296-15GL002919	43.84	2.00E-122	mechanism of antibiotic resistance|determinant of antibiotic resistance|antibiotic molecule	CmeB is the inner membrane transporter the CmeABC multidrug efflux complex. [PMID:15728904]
4	88296-15GL002994	42.73	1.00E-127	mechanism of antibiotic resistance|determinant of antibiotic resistance|antibiotic molecule	Pleuromutilin (Tiamulin) ABC efflux pump found in Paenibacillus sp. LC231, a strain of Paenibacillus isolated from Lechuguilla Cave, NM, USA. Confers resistance to pleuromutilin antibiotics. Described by Pawlowski et al. 2016. [PMID:27929110]
5	88296-15GL003296	50.13	0	antibiotic target|mechanism of antibiotic resistance|determinant of antibiotic resistance|antibiotic molecule	Point mutations that occurs in Staphylococcus aureus rpoC resulting in resistance to daptomycin [PMID:16723576]
6	88296-15GL003298	45.87	0	antibiotic target|mechanism of antibiotic resistance|determinant of antibiotic resistance|antibiotic molecule	Point mutations that occurs in Escherichia coli rpoB resulting in resistance to rifampicin [PMID:3050121]
7	88296-15GL003318	69.47	2.00E-159	antibiotic target|mechanism of antibiotic resistance|determinant of antibiotic resistance|antibiotic molecule	Sequence variants of Enterococcus faecium elongation factor Tu that confer resistance to GE2270A [PMID:7989561]
8	88296-15GL003327	41.26	1.00E-39	mechanism of antibiotic resistance|determinant of antibiotic resistance|antibiotic molecule	ArlR is a response regulator that binds to the norA promoter to activate expression. ArlR must first be phosphorylated by ArlS. [PMID:10633099]
9	88296-15GL003399	78.57	6.00E-57	mechanism of antibiotic resistance|determinant of antibiotic resistance|antibiotic molecule	catB8 is a plasmid or integron-encoded variant of the cat gene found in Klebsiella pneumoniae, Salmonella typhi and Pseudomonas aeruginosa [PMID:12760886]
10	88296-	40.94	6.00E-64	antibiotic target|mechanism of	Specific mutations on the Mycobacterium tuberculosis

	15GL003401			antibiotic resistance|determinant of antibiotic resistance|antibiotic molecule	kasA gene resulting in lowered affinity of isoniazid, resulting in resistance [PMID:10428945, PMID:9616124]
11	88296-15GL003716	41.57	6.00E-25	mechanism of antibiotic resistance|determinant of antibiotic resistance|antibiotic molecule	bcrA is an ABC transporter found in Bacillus licheniformis that confers bacitracin resistance [PMID:7476193]
12	88296-15GL003754	45.08	2.00E-54	antibiotic target|mechanism of antibiotic resistance|determinant of antibiotic resistance|antibiotic molecule	fabG is a 3-oxoacyl-acyl carrier protein reductase involved in lipid metabolism and fatty acid biosynthesis. The bacterial biocide Triclosan blocks the final reduction step in fatty acid elongation, inhibiting biosynthesis. Point mutations in fabG can confer resistance to Triclosan. [PMID:27577999]
13	88296-15GL004177	40.83	2.00E-26	mechanism of antibiotic resistance|determinant of antibiotic resistance|antibiotic molecule	ArlR is a response regulator that binds to the norA promoter to activate expression. ArlR must first be phosphorylated by ArlS. [PMID:10633099]
14	88296-15GL004342	52.07	7.00E-66	antibiotic target|mechanism of antibiotic resistance|determinant of antibiotic resistance|antibiotic molecule	fabG is a 3-oxoacyl-acyl carrier protein reductase involved in lipid metabolism and fatty acid biosynthesis.The bacterial biocide Triclosan blocks the final reduction step in fatty acid elongation, inhibiting biosynthesis. Point mutations in fabG can confer resistance to Triclosan. [PMID:27577999]
15	88296-15GL005088	72.29	4.00E-33	antibiotic target|mechanism of antibiotic resistance|determinant of antibiotic resistance|antibiotic molecule	dfrE is a chromosome-encoded dihydrofolate reductase found in Enterococcus faecalis [PMID:9869579]
16	88296-15GL005449	42.01	2.00E-25	mechanism of antibiotic resistance|determinant of antibiotic	farA is the membrane fusion protein that is part of the farAB efflux pump. [PMID:10447892]

17	88296-15GL006031	46.07	2.00E-46	resistance|antibiotic molecule mechanism of antibiotic resistance|determinant of antibiotic resistance|antibiotic molecule	TLA-1 is a beta-lactamase found in plasmids of clinical isolates of Escherichia coli strain R170 in Latin America. It preferentially hydrolyzed cephaloridine, cefotaxime, cephalothin, benzylpenicillin, and ceftazidime. The enzyme was markedly inhibited by sulbactam, tazobactam, and clavulanic acid. [PMID:15095209]
18	88296-15GL006890	59.26	4.00E-30	mechanism of antibiotic resistance|determinant of antibiotic resistance	AbeS in an efflux pump of the SMR family of transporters found in Acinetobacter baumannii. [PMID:19770280]
19	88296-15GL007254	40	1.00E-13	mechanism of antibiotic resistance|determinant of antibiotic resistance|antibiotic molecule	arr-1 is a chromosome-encoded ribosyltransferase found in Mycobacterium smegmatis [PMID:9371349]
20	88296-15GL007471	50.74	5.00E-40	mechanism of antibiotic resistance|determinant of antibiotic resistance|antibiotic molecule	TLA-1 is a beta-lactamase found in plasmids of clinical isolates of Escherichia coli strain R170 in Latin America. It preferentially hydrolyzed cephaloridine, cefotaxime, cephalothin, benzylpenicillin, and ceftazidime. The enzyme was markedly inhibited by sulbactam, tazobactam, and clavulanic acid. [PMID:15095209]
21	88296-15GL007491	66.78	0	antibiotic target|mechanism of antibiotic resistance|determinant of antibiotic resistance|antibiotic molecule	Point mutation in Capnocytophaga gingivalis that decreases binding affinity of fluoroquinolone antibiotics to gyrA, thereby conferring resistance. [PMID:27531625]
22	88296-15GL008105	41.96	7.00E-104	inhibitor of antibiotic resistance|mechanism of antibiotic resistance|determinant of antibiotic resistance|antibiotic molecule	MexB is the inner membrane multidrug exporter of the efflux complex MexAB-OprM. [PMID:19361527, PMID:20583998, PMID:21178960, PMID:14973037]

23	88296-15GL008343	43.52	6.00E-16	mechanism of antibiotic resistance|determinant of antibiotic resistance|antibiotic molecule	ykkD is an SMR-type protein that is a subunit of the ykkCD efflux pump [PMID:10735877]
24	88296-15GL008557	41.36	5.00E-37	antibiotic target|mechanism of antibiotic resistance|determinant of antibiotic resistance|antibiotic molecule	dfrA3 is an integron-encoded dihydrofolate reductase found in Escherichia coli [PMID:20169085]
25	88296-15GL008565	43.64	7.00E-29	mechanism of antibiotic resistance|determinant of antibiotic resistance|antibiotic molecule	VanG is a D-Ala-D-Ala ligase homolog that can synthesize D-Ala-D-Ser, an alternative substrate for peptidoglycan synthesis that reduces vancomycin binding affinity in Enterococcus faecalis [PMID:16323116, PMID:12426332]

Reference

1. Hantsis-Zacharov E, Shakéd T, Senderovich Y, Halpern M. 2008. *Chryseobacterium oranimense* sp. nov., a psychrotolerant, proteolytic and lipolytic bacterium isolated from raw cow's milk. Int J Syst Evol Microbiol 58:2635–2639.
